# Mice monitor their timing errors

**DOI:** 10.1038/s41598-024-71921-2

**Published:** 2024-10-07

**Authors:** Tutku Öztel, Fuat Balcı

**Affiliations:** 1 Department of Psychology, Koç University, Istanbul, Turkey; 2 Research Center for Translational Medicine, Koç University, Istanbul, Turkey; 3 Department of Biological Sciences, University of Manitoba, 50 Sifton Road, Winnipeg, MB R3T 2M5 Canada

**Keywords:** Temporal error monitoring, Higher order cognition, Animal behavior, Interval timing, Response rate, Experimental evolution, Psychology

## Abstract

Animals often engage in representationally guided goal-directed behaviors. These behaviors are thus also subjected to representational uncertainty (e.g. timing uncertainty during waiting), which has been previously shown to adaptively guide behaviors normatively. These observations raise the question of whether non-human animals can track the direction and magnitude of their timing errors (i.e. temporal error monitoring). Only a few studies have investigated this question without addressing the key components of temporal error monitoring (e.g. due to differential reinforcement of metacognitive judgments and primary task representation). We conducted the critical test of temporal error monitoring in mice by developing a novel behavioral task that involved temporal production that exponentially favored temporal accuracy and minimized the contribution of sensorimotor noise. The response rate for an upcoming probabilistic reward following the timing performance was used as a proxy for confidence. We found that mice exhibited high reward expectancy after accurate and low reward expectancy after inaccurate timing performance. The reward expectancy decreased as a function of deviations from the target interval for the short and long reproductions; pointing to the symmetrical sensitivity of metacognition to shorter/longer than target responses. These findings suggest a complete temporal error monitoring ability for mice with human-like metacognitive features.

## Significance statement

Humans tend to monitor their timing errors (e.g. whether they have overshot or undercut the recommended 45 s of wall-sitting), which guide the refinement of actions. Although other animals possess a similar timing mechanism, whether they demonstrate such higher-order cognitive processing of metric errors is part of an important debate in both behavioral and neurosciences. We tested this question by investigating how the reward expectancy of mice is modulated with the magnitude of their own timing errors in a temporal reproduction task. Our results demonstrate for the first time that mice could monitor the magnitude of their temporal errors pointing at a rudimentary form of metacognition in non-human animals.

## Mice monitor their timing errors

Animals constantly engage in goal-directed actions shaped by multiple factors, including environmental statistics (task parameters in experımental settings) and the internal state of the agent (e.g. expectancy and utility of outcomes). Often, such goal-directed actions are subject to perturbations originating from the sensorimotor system or the uncertainty in the representation of task parameters resulting in deviations from the aimed endpoints (e.g. an archerfish making an angular error in a water jet shot at prey). Although it is known that the brain adaptively responds to observed metric deviations from the target (e.g. Ref.^1^), presumably to help improve the accuracy of future actions, the extent to which agents can monitor the magnitude of unobserved deviations from the target has not been widely investigated until recently. To this end, various studies have shown that humans can monitor the direction and magnitude of errors in their timing, counting and spatial estimations (e.g. Refs.^2–9^). Precisely, humans can match their relative confidence to the amount of error (i.e. the magnitude) as reflected in an inverse relationship between error magnitude and the confidence ratings (e.g. Refs.^2,6^) and better than chance, identify whether their estimations were smaller/shorter or larger/longer than the target magnitude.

One pending question is whether non-human animals can monitor their errors without explicit feedback. This question has been investigated in nonhuman animals in various cognitive domains. For example, Kornell et al.^10^ found that monkeys express their level of uncertainty through information-seeking behavior. In line with this, Smith and colleagues^11^ demonstrated that monkeys express their level of uncertainty by opting out of deciding in difficult trials more than easier ones. Critically, this behavior persisted even in the absence of reward, demonstrating that the uncertainty responses of monkeys cannot be explained by the differential reward association with different trial types. These results indicate higher-order processing resembling metacognitive monitoring in humans (see also Refs.^12,13^). Similarly, Kirk and colleagues^14^ demonstrated that rats sought cues for the location of the large reward even after the reward was removed in a T-maze, and the information-seeking behavior generalized to the eight-arm maze where cues carried higher information value.

A few studies have also tested whether the error monitoring ability of animals generalizes to timing behavior. In an early study, Foote and Crystal^15^ tested rats in a temporal discrimination task and provided an opt-out option that led to a small but guaranteed reward. They found that rats chose the opt-out option more frequently for test durations between the short and long reference durations. However, since the opt-out option was reinforced, what appeared as uncertainty monitoring could be a choice behavior shaped by differential reinforcement. Two recent studies directly tested whether non-human animals could monitor their timing errors. Kononowicz and colleagues^16^ trained rats to produce at least 3.2 s to potentially receive a reward based on the accuracy of their subsequent actions. After each production, rats claimed the reward by choosing one of the reward ports. One of these ports provided a larger reward for small errors (port A), while the other provided smaller rewards for large errors (port B). Accordingly, subjects received two pellets for choosing port A for small errors, while no pellets were provided from this port for large errors. Similarly, subjects received one pellet for choosing port B for large errors, which provided no reward for small errors. Results demonstrated that rats could correctly discriminate the magnitude of their timing errors. However, this work used a crude mapping between first and second-order responses using a two-alternative forced choice paradigm, where only errors longer than the target were investigated.

One way to address this shortcoming is to investigate the relationship between the temporal errors and the reward expectancy (measured through the response rate). Accordingly, the response rate is expected to be an inverse function of the error amount. More recently, Minary^17^ used a similar timing task with a critical modification. The response window of a variable duration was introduced after each timing response under the assumption that the response rate during this response window would serve as a proxy for the level of reward expectancy and, thus, confidence in the relative accuracy of the timing response. They found that an exponential-Wald mixture distribution best accounted for timing responses; where the exponential portion captured impulsiveness and the Wald portion captured the cognitively-controlled responses. Notably, the response rate recorded during the variable response window was lower after impulsive responses and higher after the cognitively controlled responses, which points to the idea that mice can discriminate their correct versus incorrect reproductions. Thus, this result is in line with the earlier findings of Kononowicz et al.^16^.

Both Kononowicz et al.^16^ and Minary et al.^17^ trained animals to produce a minimum duration to receive a reward. Resultantly, their subjects responded to maximize reward rate based on a hidden step function of reward availability (transitioning from zero to one at the minimum duration requirement), which has been robustly shown to lead to a reward rate maximizing adaptive bias in timing behavior. Specifically, in tasks with minimum duration requirement, animals tend to produce time intervals longer than the minimum requirement, taking near normative account of their endogenous timing uncertainty for maximizing reward rate (e.g. Refs.^16,18,19^). Crucially, the step-function based reward function used in these tasks nevitably introduces a meta-task representation (overlaid on the first-order timing responses) that relies on two different cognitive states (cognitive control vs. impulsivity). Thus, any form of error monitoring demonstrated by animals might partially reflect their ability to differentiate between impulsivity and cognitive control (in tasks that require cognitive control but result in a mixture of impulsive and cognitively controlled responses) rather than monitoring the degree of their timing errors as a necessary conceptual element for claiming temporal error monitoring. To this end, Minary ^17^ showed that although mice have lower reward expectancy following impulsive responses, the level of expectancy does not correlate with deviations from the target duration for cognitively controlled responses.

We conducted the critical test of temporal error monitoring in mice by training them to produce the target interval as accurately as possible based on a double exponential reward function (favoring longer but close to the target duration). This modification of the previous paradigms^16,17^ minimized the timing behavior biases shaped by earlier task representations.

Accordingly, if mice could monitor the magnitude of their temporal errors, their reward expectancy behavior should be maximal for the reproductions around the mean/target and diminishing for reproductions that increasingly deviate from the target. Similarly, if the error awareness of mice was sensitive to both “earlier errors” and “longer errors”, the reward expectancy behavior should be symmetrical for reproductions shorter and longer than the target durations.

## Materials and methods

### Subjects

Seven naive male C57BL6 mice (bred at Koç University Research Center in Translational Medicine) were used in the experiment. Behavioral training started when mice were 8–10 weeks old. Mice were group-housed in groups of four mice per cage in a 12 h light cycle. The sessions were conducted for an hour in the light cycle starting from 7:30 AM every weekday. The subjects' weight was kept at 85–95% of their ad-lib weight through a food restriction regime. Water was always freely available in their home cages. All the experimental procedures were approved by the Koç University Animal Research Local Ethics Committee (ethical approval number: 2016.034) and the study was conducted in accordance with the relevant guidelines and regulations. This study was not preregistered. All the methods and procedures were reported in accordance with ARRIVE guidelines^20^.

### Apparatus

The experiment was conducted in operant chambers (21.6 cm × 17.8 cm × 12.7 cm, ENV-307W; Med Associates) placed in sound-attenuated cubicles (40 cm × 69 cm × 41.5 cm). Each operant chamber had two metal walls. Two retractable levers and a central hopper were placed on one of the walls, while three hoppers with IR beam break detectors and a house light were placed on the opposite wall. The liquid reward was delivered at a 0.01 mL volume in the middle hopper. The reward was a liquid food prepared as a mixture of tap water and powdered coffee creamer (1:1) kept at 4 °C. The hardware was controlled, and data were recorded with MedPC.

### Procedure

#### Fixed ratio 1-fixed time 60 (FR1-FT60): Phase 1)

The FR1-FT60 (Phase 1) familiarized the mice with lever pressing behavior and the operant chambers. Each trial started with the insertion of the active lever and the illumination of the houselight. If the subject pressed the lever within 60 s, the central reward hopper was illuminated, and the reward was presented (available for 6 s). If, on the other hand, the subject did not press the lever within 60 s, the houselight was turned off; the lever was retracted and the reward was nevertheless presented after 60 s (i.e. autoshaping). The reward was always available for 6 s. The active lever was counterbalanced across mice. The FR1-FT60 phase lasted for three consecutive sessions.

#### Fixed ratio 1 (FR1): Phase 2

The FR1 was the same as the FR1-FT60 phase, except mice received the reward only after pressing the lever where the lever was available until a response had been made by the subject. Mice moved to the next phase (Phase 3) after they pressed the lever a minimum of forty times for two consecutive days.

#### Reproduction and reward expectancy: Phase 3

**Fig. 1:**
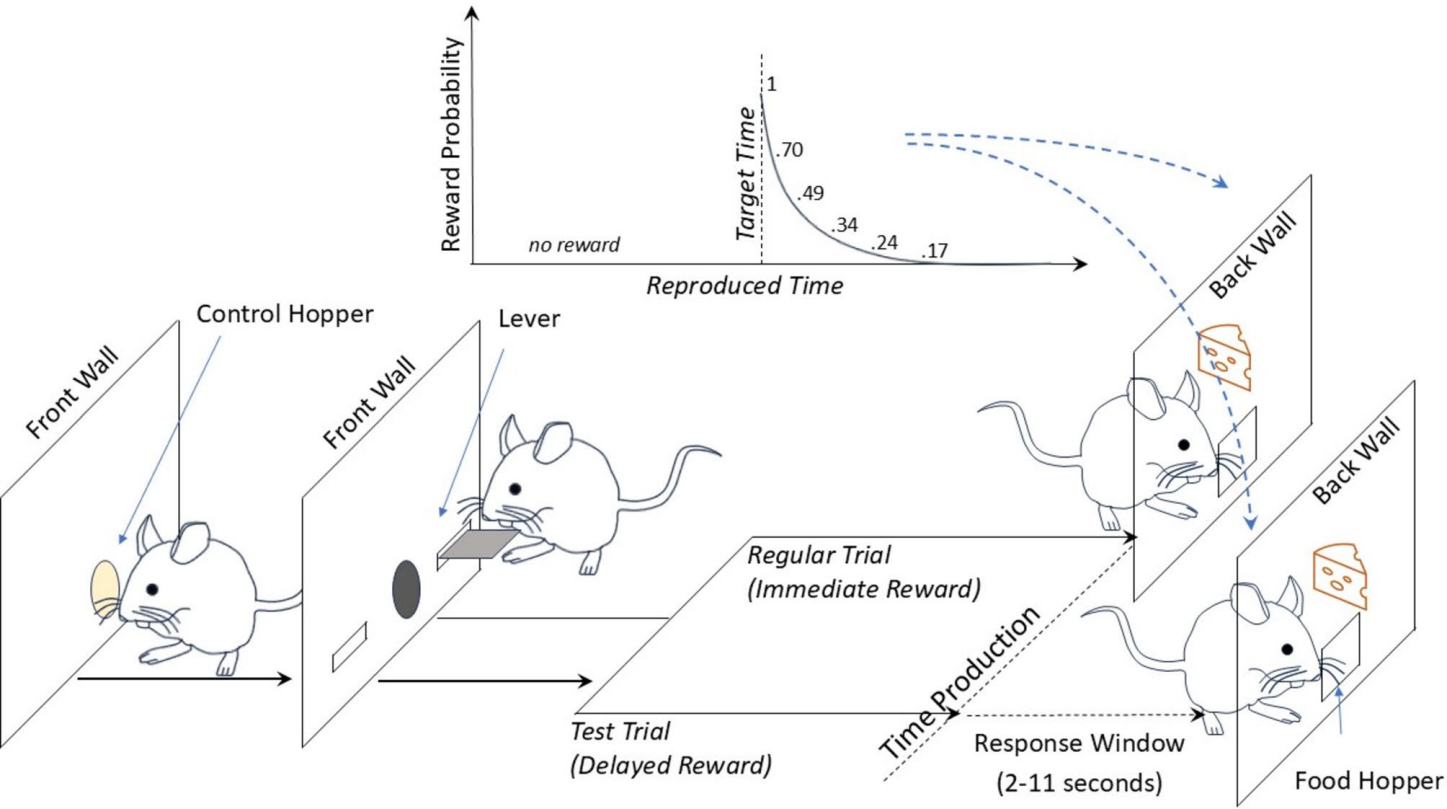
The graphical illustration of the Phase 3. Mice initiate the trials by nose-poking in the central control hopper. Either of the levers inserted upon the trial initiation is depressed by the animal for a duration (referred to as time production). If the trial type is “training”/regular, the animal is rewarded instantly according to the reward probability scheme for their temporal reproductions. If the trial type is “test,” then the animal is rewarded according to the reward probability scheme after a randomly chosen delay between 2 and 11 s.

All trials were initiated by mice upon nose poking into the illuminated central control beam, upon which the house light was turned on. There were two trial types. In training trials, mice were immediately reinforced with a geometrically deccreasing probability, which was maximal at the target interval (where for each target/11 ms of bin; *p*(reward|reproduction) =  ~ [1, 0.7, 0.49, 0.34, 0.24, 0.17]). In test trials, the reward was delivered according to the same rule but only after a 2–11 s long response window during which mice typically nose poked into the hopper in anticipation of the reward delivery. In neither of the trial types, the reproductions shorter than the target duration were reinforced, which resulted in the immediate termination of the trial with a retracted lever and unlit central food hopper. All mice (except for one) were trained to reproduce a target duration of 550, 750 or 1100 ms by depressing the lever, where they proceeded to the next target duration after completing training with the shorter target. One mouse was trained up to 2200 ms. Figure 1 illustrates the procedure of Phase 3.

## Analytical approach

### Nosepoke as confidence

Before the analysis, we calculated the nose poke rate (number of responses/feeder active duration) as an index of subjects’ reward expectancy. Higher nosepoke rates depict high reward expectancy, which we utilized as a proxy of animals’ confidence.

### Z-score transformed temporal reproductions

To capture the reproductions' directionality and magnitude, we z-score transformed the reproductions of individual mice.

### Linear mixed-effects analysis

To investigate the quadratic relationship between the temporal reprodıctions and nose poke rate in a single model, we tested our hypothesis in a linear mixed effects model as depicted below:

Model 1. Nosepoke rate ~ Temporal Reproduction^2^ + (1|subject) where ~ reads as “predicted from” and 1|subject reads as “random intercept across subjects.” The z-score temporal rep.

The model included all lower terms. We used the restricted maximum likelihood (REML) method for parameter estimations.

## Results

We first compared the coefficient of variation (CV) of the reproduced durations in training and test trials. The paired sample *t*-test revealed no significant difference between training and test trials in terms of reproduction CVs (*t*(6) = − 0.61, *p* = 0.57; *M*_test_ = 0.31, *SD*_test_ = 0.068; *M*_training_ = 0.33, *SD*_training_ = 0.094).

The mixed model revealed a significant negative quadratic relationship between the temporal reproductions and nose poke rate (ß = − 0.00646, *SE* = 0.00193, *p* < 0.001, 95% CI = [− 0.0103 − 0.00267]). As expected, the main effect of temporal reproduction on the nose poke rate was not statistically significant (ß = − 0.00181, SE = 0.0044, *p* = 0.681, 95% CI [− 0.0104 0.0068]). The negative quadratic relationship, thus, points to an increased reward expectancy for the minimal deviations in the reproduced durations. Furthermore, reward expectancy demonstrated a double-sided decrease for the reproductions with high symmetrical deviations for short and long reproductions.

We tested whether our conclusion holds when we applied the data filtering method as practiced with human participants (i.e. discarding trials with three standard deviations (SD) above the mean of the reproductions; for a detailed discussion, see: Öztel & Balcı, 2024^21^). When trials with reproductions three SD above the mean were discarded, there was a statistical trend for a quadratic relationship between the temporal reproductions and nosepoke rate (*p* = 0.079). Given that this practice could be overly conservative for animal subjects given their much smaller temporal precision (average coefficient of variation = 0.63 + − 0.01; where coefficient of variation is calculated as *SD*/*M*^22^), we also applied a more liberal data filtering method (five SD instead of three SD). This practice yielded a preserved quadratic relationship between the temporal reproductions and nosepoke rate (ß = − 0.007, *SE* = 0.002, 95% CI = [− 0.011 − 0.0022], *p* = 0.0033).

**Fig. 2:**
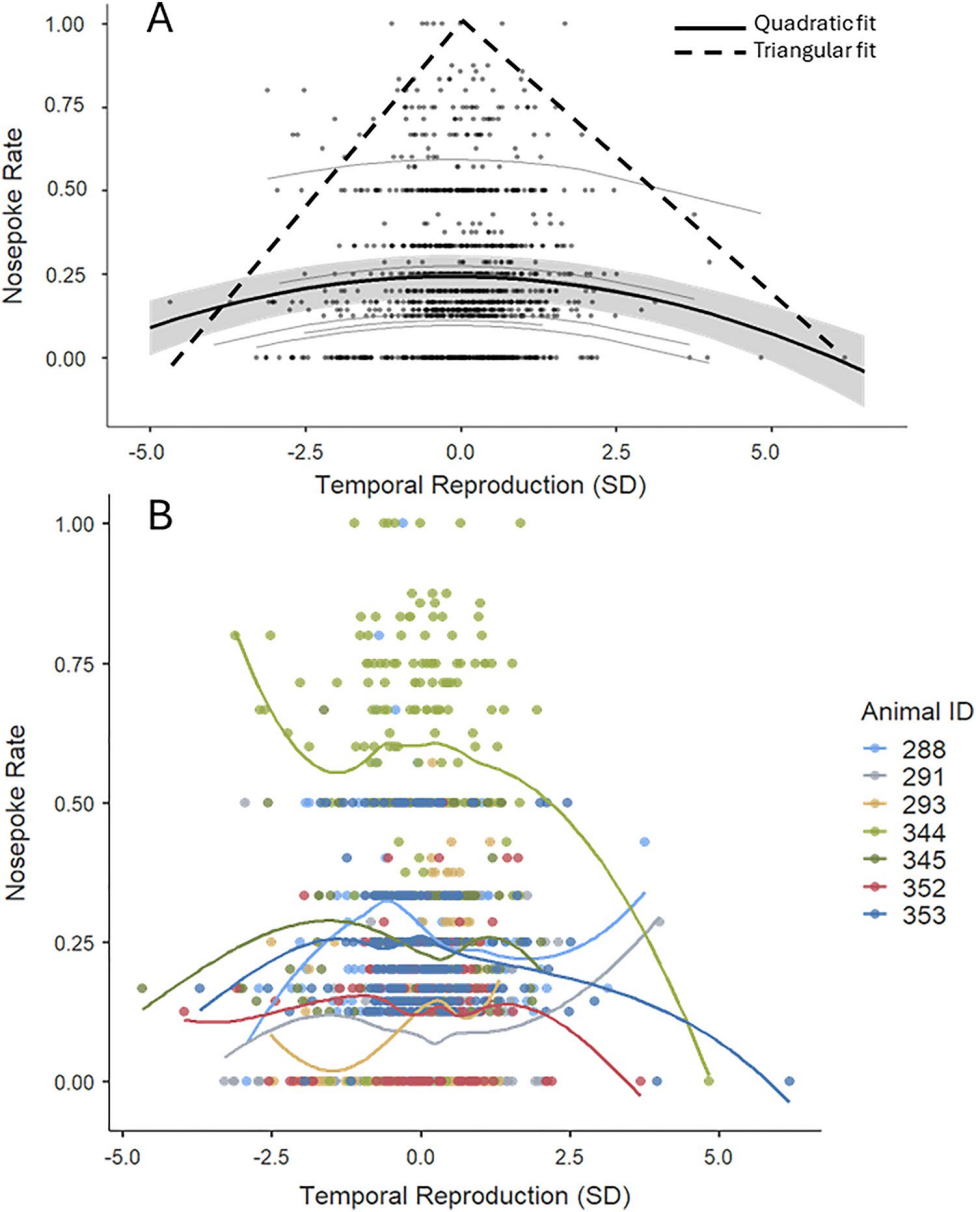
(**A**) The curvilinear relationship between z score transformed temporal reproductions and nose poke rates and the associated triangular fit. The shaded gray area depicts the standard error of the estimate (SE), the scatter plots depict individual data points, and the thin gray lines depict individual regression lines of each subject. Triangular distribution fit to the minimum, median and maximum values of temporal reproductions. (**B**) The scatter plots and smoothed regression lines for each mouse.

We also fit a triangular distribution to the temporal reproductions based on the minimum, median, and maximum values. The triangular distribution approximated the higher response rate for accurate temporal productions. Figure 2A illustrates the quadratic relationship between temporal reproductions and nose poke rates, along with the associated triangular fit (goodness of fit (in error norm) = 0.987 with associated cost function of Mean Squared Error (MSE); individual data is illustrated in Fig. 2B).

## Discussion

It has long been documented that animals emit goal-directed behaviors shaped by exogenous (e.g. delay to reward, magnitude of reward) and endogenous states (e.g. level of uncertainty, incentive motivation). For instance, humans and other animals can adopt a degree of bias (considering the uncertainty in their timing and counting behaviors) to maximize the reward rate attained in a task (e.g.Refs.^18,19^). The cognitive penetrability of goal-directed behaviors also raises the unresolved question of whether animals can ¨report¨ their confidence in the accuracy of such behaviors based on the reliability of the information that guides them. Previous research demonstrated that nonhuman animals could demonstrate more frequent cue-seeking behavior (e.g. Refs.^10,14^) before making a decision about stimuli with a low signal-to-noise ratio in perceptual decision-making tasks. Despite task-dependent limitations, recent work suggested that rodents can also monitor the degree of their timing errors that result from endogenous timing uncertainty^16,17^. The current work aimed to investigate whether mice can monitor both the degree of their timing errors in both directions (earlier and longer than target) based on a novel behavioral task that exponentially favored accurate timing behavior in a few seconds range.

We found that the level of reward expectancy decreased with increasing deviation from the target interval in both directions. This relation was well-captured by a negative quadratic function mapping the nosepoke rates during the response window onto the directional temporal reproductions. These results provide both direct and robust evidence for the temporal error monitoring abilities that could capture both the shorter and longer than target timing errors^16,17^ see also Ref.^15^).

The previous studies by Kononowicz et al.^16^ and Minary et al.^17^ did not sufficiently address the question of whether rodents can monitor earlier than the target errors (Minary^17^ did not address error magnitude, either) due to the step-function-based reward availability favoring response times much-longer than target durations. Furthermore, the choice tasks devised by Kononowicz et al.^16^ and Foote and Crystal^15^ required differential reinforcement of second-order judgments rather than relying on an ecologically valid and implicit measure for reward expectancy (i.e. response rate). On these grounds, the results of the current study provide a more comprehensive and confound-free handling of temporal error monitoring ability as it has been studied in human participants (e.g. Refs.^2,6,9^; for a detailed discussion).

Note that ^22^different from these earlier studies, the procedure we developed for this study resulted in highly accurate and precise timing behaviors even for very short intervals (500–2500 ms). The added gain of our work is that it provides methodological tools to study few-seconds long interval timing in mice without contamination by task-dependent large adaptive biases or non-timing specific sensorimotor noise.

One limitation of the current study is that it is possible that the mice learned the hidden exponential reward structure and aimed for specific time points based on the recalled associated reward probability (instead of aiming for the highest earning target interval in each trial). But in this case, we would expect higher than usual CV values. Thus, we assert that the modulation of the response rates, guided by the underlying reward geometry, would require the animal to know what their temporal reproductions are and, thus, metacognitive processing. Another limitation of the current study is that the two distinct components (i.e. the magnitude and direction monitoring) are investigated within the same response rate variable in a composite form. This approach does not allow the investigation of the idiosyncratic characteristics of these two components of error monitoring that have been recently demonstrated to have distinct features for human adults^23^. To achieve a more granular analysis of temporal error monitoring performance, future studies should devise a paradigm that can capture the distinct features of the two components for nonhuman animals.

Overall, our results point to a behavioral pattern that suggests higher-order cognitive processing in mice. Concerning the previous work^15–17^, the response rate measure used in the current study constitutes a more holistic way of evaluating the metacognitive architecture of confidence reporting in the form of reward expectancy. Future studies should devise Future studies should also investigate whether similar patterns could be observed in the numerical and spatial metric domains. A potential parallelism between different quantitative domains could reveal a domain-general metacognitive system (e.g. also see Refs.^9,24^).

## Conclusion

The current study investigated whether mice can monitor the magnitude of their timing errors (both earlier and later than target errors), which is called temporal error monitoring^2^. In line with the temporal error monitoring hypothesis, we found a negative quadratic relationship between the temporal errors and response rates. This suggests that mice can keep track of the magnitude of their self-generated temporal errors by symmetrically matching their response rate to the amount of their timing errors. This ability might be the cognitive factor that mediates the optimization of timing and numerical responses in light of timing and numerical uncertainty, respectively (e.g. Refs.^18,19,25^). These results suggest that error monitoring is an evolutionarily well-preserved ability, at least when considering humans, rats, and mice.

## Data Availability

All data and analysis materials will be available upon request (contact: toztel17@ku.edu.tr; fuat.balci@umanitoba.ca).
